# Paleoneuroanatomy of the aetosaur *Neoaetosauroides engaeus* (Archosauria: Pseudosuchia) and its paleobiological implications among archosauriforms

**DOI:** 10.7717/peerj.5456

**Published:** 2018-08-22

**Authors:** M. Belen von Baczko, Jeremías R.A. Taborda, Julia Brenda Desojo

**Affiliations:** 1 División Paleontología de Vertebrados, Museo de La Plata, La Plata, Argentina; 2 Consejo Nacional de Investigaciones Científicas y Tecnológicas (CONICET), Ciudad Autónoma de Buenos Aires, Argentina; 3 Centro de Investigaciones en Ciencias de la Tierra (CICTERRA), Universidad Nacional de Córdoba - CONICET, Córdoba, Argentina

**Keywords:** Neuroanatomy, Triassic, Paleobiology, Archosauriforms, Aetosauria

## Abstract

The paleoneuroanatomy of pseudosuchian archosaurs is poorly known, based on direct examination of the internal morphology of braincases and a few artificial endocasts. Among aetosaurs, only one endocast has been described almost a century ago by Case (1921) corresponding to *Desmatosuchus spurensis* from the Chinle Formation (Norian) of Texas, US, based on a resin cast. Here, we describe the first natural endocast of an aetosaur, *Neoaetosauroides engaeus* from the Los Colorados Formation (Norian) of NW Argentina, and also developed the first digital endocast of this taxon including the encephalon, cranial nerves, inner ear, and middle ear sinuses. The neuroanatomy of *Neoaetosauroides engaeus* exhibits several differences from that of *Desmatosuchus spurensis* despite their phylogenetic proximity, which may be a reflection of their different habits. The information provided by the endocasts of *Neoaetosauroides engaeus* about its olfactory region and the orientation of its head, based on the inclination of the inner ear, could support the proposal for an animalivorous diet, instead of an herbivorous one as in most aetosaurs. The new information here obtained contributes to the knowledge of the neuroanatomical diversity of archosauriforms and more specifically among pseudosuchians and their paleobiological roles in the Triassic continental communities.

## Introduction

Aetosaurs are a group of terrestrial quadrupedal archosaurs, with body sizes ranging from one to five metres long, recorded from the Late Triassic of America, Europe, Africa, and Asia. They are characterized by armored bodies composed of dorsal, ventral, and appendicular ornamented osteoderms (Heckert & Lucas, 2000; Desojo et al., 2013; Parker, 2016), small, triangular, skulls with expanded shovel shaped premaxillae (excepting *Aetosauroides scagliai*, *Aetosaurus ferratus*, and *Paratypothorax andressorum*), and dentaries with a peculiar shape, being anteriorly concave, edentulous, and tapering distally (excepting *Aetosauroides scagliai*) (Desojo & Ezcurra, 2011; Schoch & Desojo, 2016). Aetosaurs have been traditionally considered as the only herbivorous pseudosuchians; however, the diversity of their dental morphology allowed new interpretations on the diet of this group, and some authors suggested animalivorous habits for the basal-most aetosaurs such as *Neoaetosauroides engaeus*, *Aetosauroides scagliai*, and *Aetosaurus ferratus* (Desojo & Báez, 2007; Desojo & Vizcaíno, 2009). This group has been historically considered as index fossils for the Late Triassic (e.g. Heckert & Lucas, 1999; Heckert & Lucas, 2000; Parker, 2007; Parker, 2016) although some authors debate this utility based on several reasons such as homoplasy, ontogenetic changes, and sexual dimorphism (Taborda, Cerda & Desojo, 2013; Taborda, Heckert & Desojo, 2015; Parker, 2016; Schoch & Desojo, 2016).

Even though the external anatomy of aetosaurian skulls is well known, only one endocast was described in detail almost a century ago by Case (1921). This description of the encephalon of the aetosaur *Desmatosuchus spurensis* was based on an artificial endocast of the cranial cavity of the holotype and represents not only the encephalon, but also the soft tissues surrounding it, such as the dura mater and venous sinuses (Hopson, 1979; Gans & Billett, 1985). In living pseudosuchians, these surrounding tissues represent approximately 50% of the endocranial cavity (Jirak & Janacek, 2017), differing from that of other living archosaurs such as birds in which the encephalon essentially fills the endocranial cavity (Hopson, 1979; Witmer & Ridgely, 2008). The study of the internal cavities of the skull (encephalon, inner ear, paranasal sinuses, cranial nerves, muscles) by applying computed tomography to fossils allowed the collection of crucial anatomical information for morphological, ontogenetic, and functional analyses (Sereno et al., 2007; Balanoff et al., 2013; Paulina-Carabajal, Carballido & Currie, 2014; Jirak & Janacek, 2017). These new studies allow a better understanding of the paleobiological roles of the different pseudosuchian groups in the Triassic continental communities worldwide.

During the redescription of the cranial anatomy of *Neoaetosauroides engaeus*, based on three skulls, the first known natural endocast of an aetosaur was discovered during the preparation of the material (Desojo, 2005; Desojo et al., 2013). This exceptionally preserved material, PULR 108, consists of the mould of the forebrain, midbrain, and hindbrain and both partially preserved inner ears. Additionally, the partial skull of *Neoaetosauroides engaeus* PVL 4363 bears the natural moulds of the olfactory tracts and bulbs *in situ*. Complementarily, the digital cast of the endocranial cavity of the most complete braincase was generated recovering the encephalon, cranial nerves, and also the first known middle ear sinuses and inner ears of an aetosaur (PVL 5698). The aim of the present contribution is to describe, analyse, and compare the endocranial anatomy of the aetosaur *N. engaeus* to comprehend the evolution of these structures within archosauriforms and its implications for paleobiology.

## Systematic Paleontology

**Table:** 

ARCHOSAURIA Cope, 1869 *sensu* Gauthier & Padian, 1985
PSEUDOSUCHIA Zittel, 1887–1890 *sensu* Gauthier & Padian, 1985
AETOSAURIA Marsh, 1884 *sensu* Parker, 2007
*NEOAETOSAUROIDES* Bonaparte, 1969
*Neoaetosauroides engaeus* Bonaparte, 1969

## Materials and Methods

### Materials

PULR 108: natural casts of inner surface of the skull, lower jaw, and natural cast of the endocranial cavity.

PVL 5698: almost complete skull lacking the snout region.

PVL 4363: incomplete skull with natural moulds of the skull roof, left side, and left jaw.

### CT scanning and digital reconstruction

The referred specimen PVL 5698 was CTscanned at the Clínica La Sagrada Familia (Buenos Aires, Argentina) using a medical 64-channel Phillips Multislice CT scanner. The dataset consists of 413 slices with the following settings: field of view 289 mm, penetration power of 120.0 Kv and 313 mA, slice thickness of 0.9 mm and 0.45 mm of overlap. Analysis of the images and 3D reconstructions were developed with the open source software 3D Slicer v4.1.1 (Fedorov et al., 2012). The terminology used for the description of the digital endocast does not refer strictly to the soft tissue regions of the brain because, as seen in modern archosaurs, the endocast also includes the volume occupied by other tissues surrounding the brain (i.e., vascular tissue) (e.g., Paulina-Carabajal & Currie, 2012; Paulina-Carabajal, Carballido & Currie, 2014).

The raw dataset is available at https://doi.org/10.6084/m9.figshare.6709289.

## Description

### Encephalon

**Figure 1:**
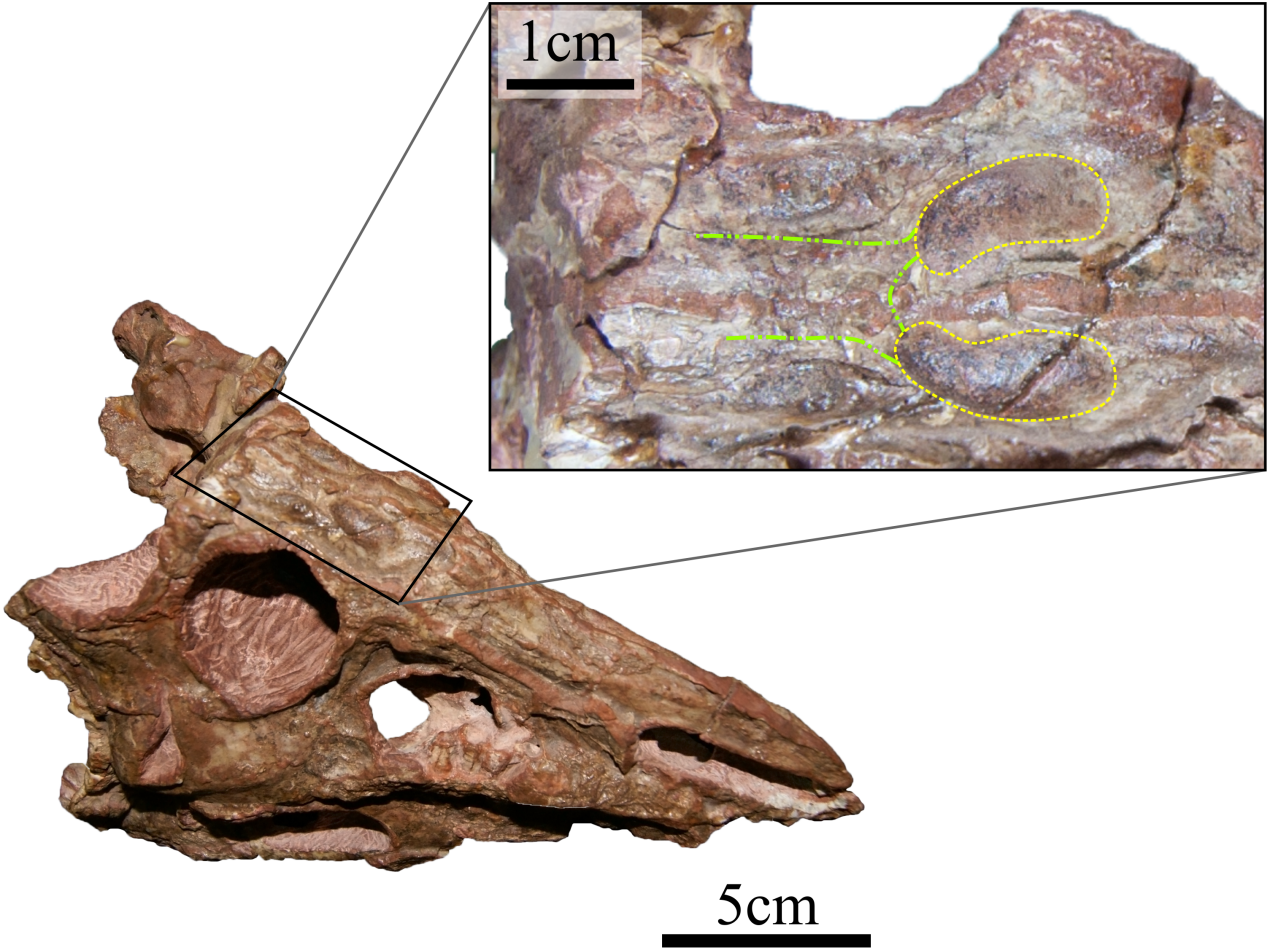
Laterodorsal view of the skull of *N. engaeus* (PVL 4363) with detail of the olfactory bulbs (yellow) and tracts (green). The specimen PVL 4363 lacks the skull roof, allowing the direct observation of the dorsal region of the natural endocast. Photographs: MB von Baczko.

**Figure 2:**
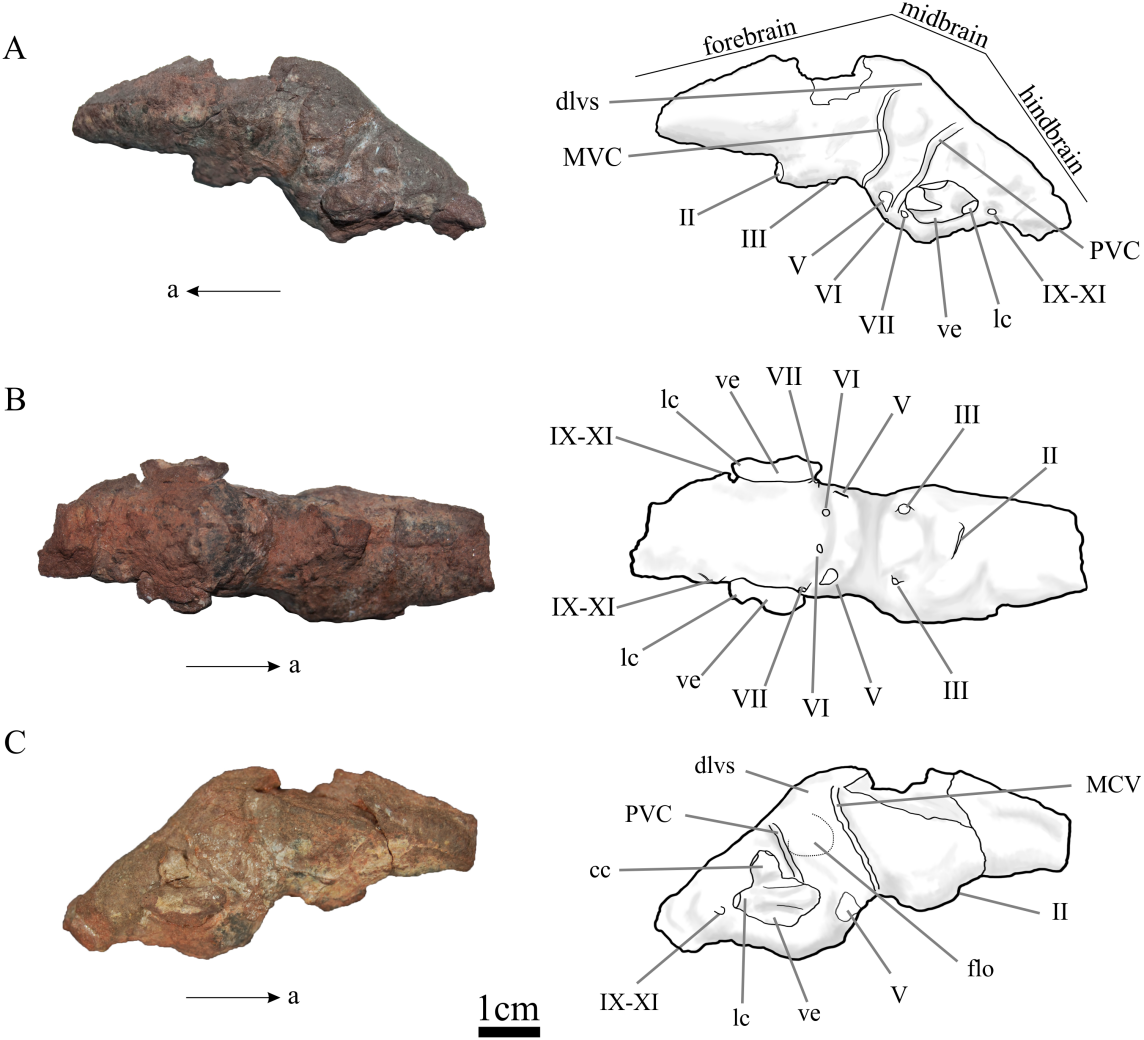
Natural endocast of *N. engaeus* (PULR 108). (A) Left view with outline; (B) ventral view with outline; (C) right view with outl ine. Abbreviations: II, cranial nerve II; III, cranial nerve III; IV, cranial nerve IV; V, cranial nerve V; VI, cranial nerve VI; VII, cranial nerve VII; VIII, cranial nerve VIII; IX–XI, cranial nerves IX, X and XI; cc, common crus; dlvs, dorsal longitudinal venous sinus; flo, flocculus; lc, lateral semicircular canal; MVC, medial vestibular constriction; PVC, posterior vestibular constriction; ve, vestibulum. Photographs: MB von Baczko. Drawings: MB von Baczko, JRA Taborda.

**Figure 3:**
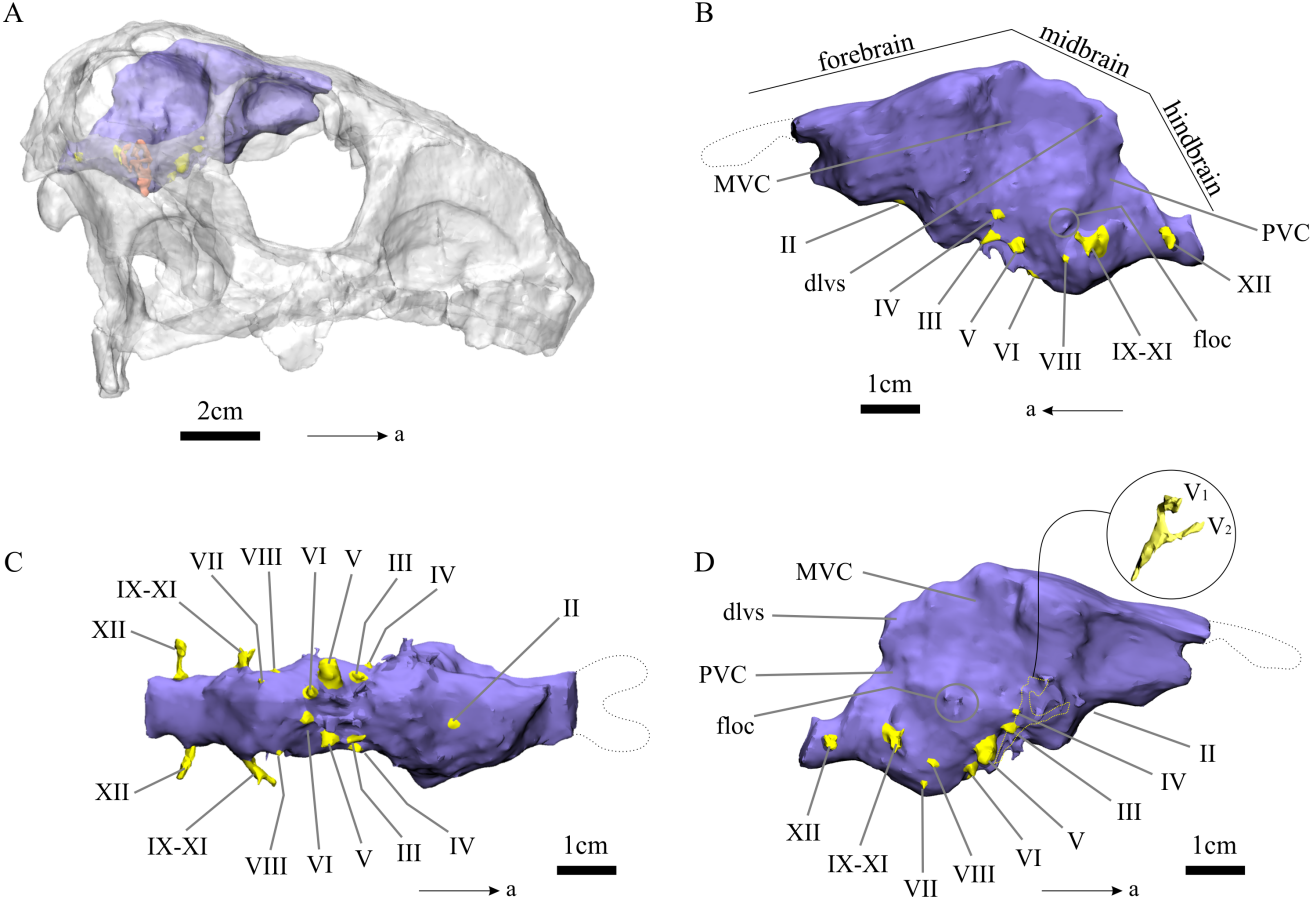
Digital endocast of *N. engaeus* (PVL 5698). (A) Endocast and right vestibular apparatus placed in its natural position in the skull in alert position. (A) Skull in dorsolateral view; digital endocast in (B) left, (C) ventral, and (D) right views. Outline of olfactory bulbs based on PVL 4363. Abbreviations: II, cranial nerve II; III, cranial nerve III; IV, cranial nerve IV; V, cranial nerve V; VI, cranial nerve VI; VII, cranial nerve VII; VIII, cranial nerve VIII; IX-XI, cranial nerves IX, X and XI; XII, cranial nerve XII; dlvs, dorsal longitudinal venous sinus; flo, flocculus; MVC, medial vestibular constriction; PVC, posterior vestibular constriction. 3D reconstruction: JRA Taborda.

The endocast of *N. engaeus* is elongate, narrow and has a strong angle between the forebrain and the midbrain (135°). The anterior margin of the olfactory bulbs is located at the level of the posterior margin of the antorbital fenestra and the posterior end is located at the level of the anteroposterior midline of the orbit (PVL 4363). The olfactory tracts are short and the bulbs are elliptic, being anteroposteriorly longer than wide. The olfactory bulbs do not contact each other at the sagittal plane (Fig. 1). The cerebral hemispheres are elongate, slightly expanded, and 1.13 times longer than wide (PULR 108). The cerebral hemispheres are the widest region of the forebrain which is 1.5 times wider than their posterior end at the medial vestibular constriction (MVC). The hypophysis should be on the ventral surface of the cerebral hemispheres, just anterior to the MVC, but it is broken on the natural cast and could not be reconstructed on the digital cast, therefore, its ventral extension is unknown. The midbrain is restricted between the MVC and the posterior vestibular constriction (PVC) (Figs. 2 and 3: MVC, PVC). It is slightly dorsally expanded, possibly because of the presence of the dorsal longitudinal venous sinus that runs anteroposteriorly from the dorsal surface of the midbrain to the hindbrain (Witmer & Ridgely, 2008) (Figs. 2A, 2C, 3B and 3D: dlvs). The floccular lobes form small lateral projections on the lateral surfaces of the midbrain (PULR 108, PVL 5698). They are represented by a hemispherical bump in lateral view and are located anterior to the vestibular apparatus (Figs. 2C, 3B and 3D: flo, ve). The hindbrain is divided from the midbrain by a flexure, but this angle cannot be clearly determined because of the presence of the dorsal longitudinal venous sinus which obscures this region of the brain.

### Cranial nerves

Most of the cranial nerves (CN) have been identified in the natural endocast of *N. engaeus* (PULR 108) and the digital reconstruction based in PVL 5698 (Figs. 2 and 3). The cranial nerves II to VI were recognized on both endocasts, but CNs VII to XII were only seen on the digital reconstruction. The CN I corresponds to the olfactory nerve indicated at the level of the olfactory tracts previously described. The optic nerve (CN II) is recognized at the level of the optic chiasma as a prominent structure located at the midline of the ventral surface of the forebrain. The oculomotor nerve (CN III) and the trochlear nerve (CN IV) are recognized on the ventral surface of the midbrain posterior to the optic chiasma and anterior to the trigeminal nerve (CN V) (Figs. 2 and 3). The oculomotor nerve is lateroventrally projected and trochlear nerve is laterally oriented. The trigeminal nerve is the largest cranial nerve and is also located on the hindbrain projecting lateroventrally. The ophthalmic and maxillary rami (CN V_1_, CN V_2_) of the trigeminal nerve could be identified on the right side of the digital endocast of *N. engaeus*, but not the mandibular branch (CN V_3_) (Fig. 3D: V_1_, V_2_). The split of the trigeminal branches apparently occurs outside the endocranial cavity (Fig. 3D: V_1_, V_2_), as also happens in most archosaurs (Witmer & Ridgely, 2008). The abducens nerve (CN VI) is placed on the ventral surface of the hindbrain ventral to the trigeminal nerve and projecting anteroventrally. The facial nerve (CN VII) also projects from the ventral surface of the hindbrain but is located posterior to the CN VI and ventromedially to the endosseous labyrinth (Fig. 3). The vestibulocochlear nerve (CN VIII) is situated posteroventrally to the CN VI and medially to the endosseous labyrinth and projects laterally.

The metotic passage can be identified posterior to the semicircular canals and would probably have accommodated the glossopharyngeal nerve (CN IX), the vagus nerve (CN X) and the spinal accessory nerve (CN XI). Two branches are recognized splitting from the metotic passage (Figs. 3B–3D). This passage can also be recognized externally as a foramen on the braincase of PVL 5698 between the ventral process of the opistotic and the lateral ridge of the exoccipital and basioccipital (*sensu* Gower & Walker, 2002). The hypoglossal nerve (CN XII) exits laterally through a single passage on the posteriormost part of the hindbrain.

### Inner ear

The inner ears of *N. engaeus* are incompletely preserved in the natural endocast of PULR 108, but were able to be reconstructed almost entirely on the right side of the digital endocast of PVL 5698 (Figs. 2 and 3A). Therefore, the following description is based only on the right inner ear of *N. engaeus*.

**Figure 4:**
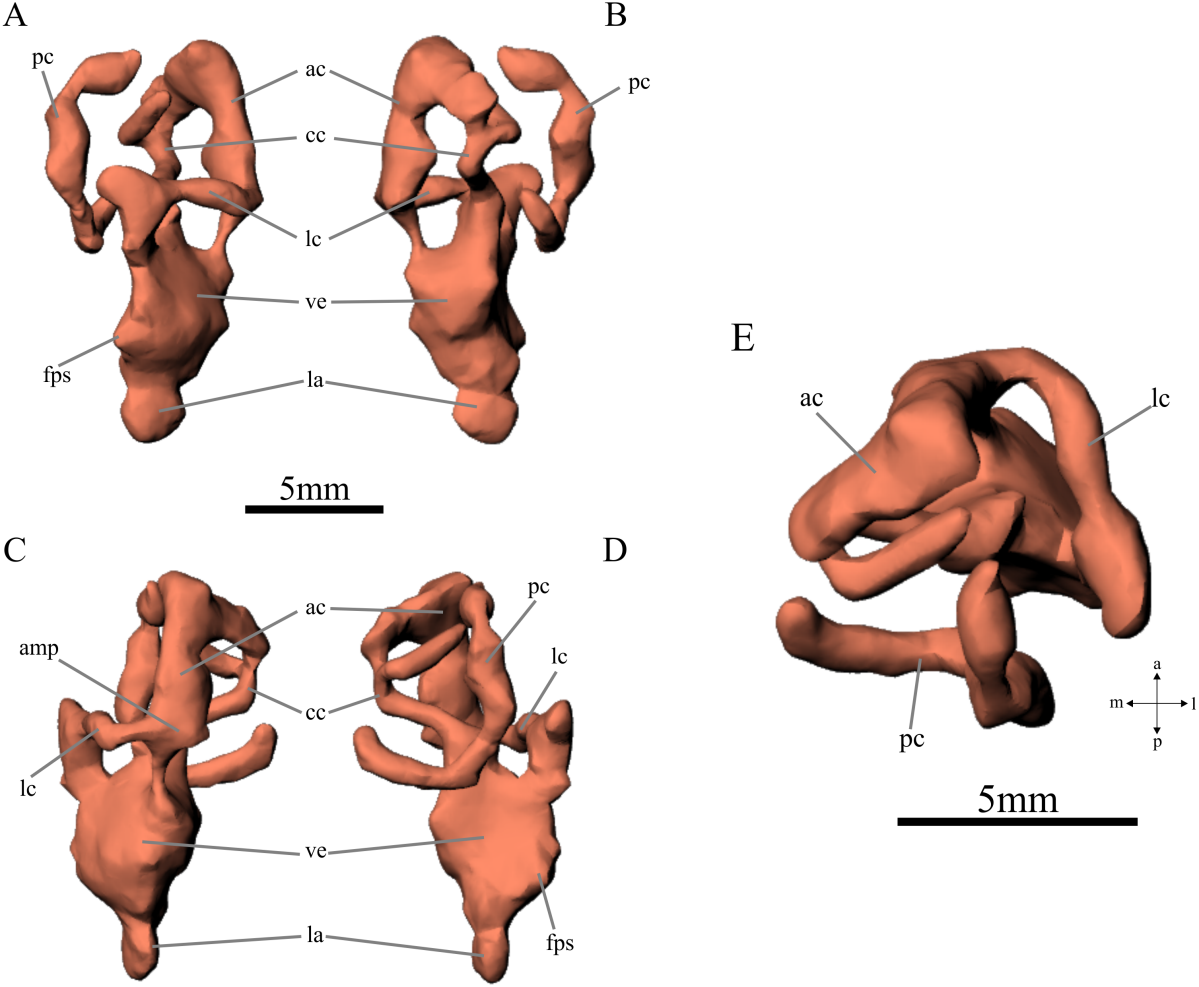
Right inner ear of *Neoaetosauroides engaeus* (PVL 5698). (A) lateral, (B) medial; (C) anterior, (D) posterior, and (E) dorsal views. Abbreviations: ac, anterior semicircular canal; amp, ampulla; cc, common crus; fps, fenestra pseudorotunda; la, lagena; lc, lateral semicircular canal; pc, posterior semicircular canal; ve, vestibulum. 3D reconstruction: JRA Taborda.

The endosseous labyrinth is as high as anteroposteriorly long, quadrangular in lateral view. The anterior and posterior semicircular canals are apparently equivalent in diameter but the anterior semicircular canal has a wider diameter of the tube. The anterior canal describes a quadrangular shape while the posterior one is more circular. These two canals form an angle of approximately 85° and connect each other dorsomedially forming a common crus that projects ventrally reaching the vestibulum (Fig. 4: ac, pc, cc, ve). The lateral semicircular canal is oval-shaped, mediolaterally compressed, and has a swelling on its contact with the anterior canal which probably represents the anterior ampula (Fig. 4: hc, amp). The posterior ampula cannot be identified because the posterior end of the lateral semicircular canal could not be reconstructed on the digital endocast. The vestibulum and the lagena were identified only on the digital reconstruction (PVL 5698). The vestibulum is located between the vestibular apparatus and the fenestra pseudorotunda; it is slightly higher than the lagena and equally long as high in lateral view (Fig. 4: fps, ve, la). The fenestra pseudorotunda is placed in the posteroventral corner of the vestibulum and the lagena is located ventral to this structure. The lagena is short, mediolaterally compressed, it maintains its anteroposterior width along its length, and is rounded distally.

### Pneumatic sinuses

**Figure 5:**
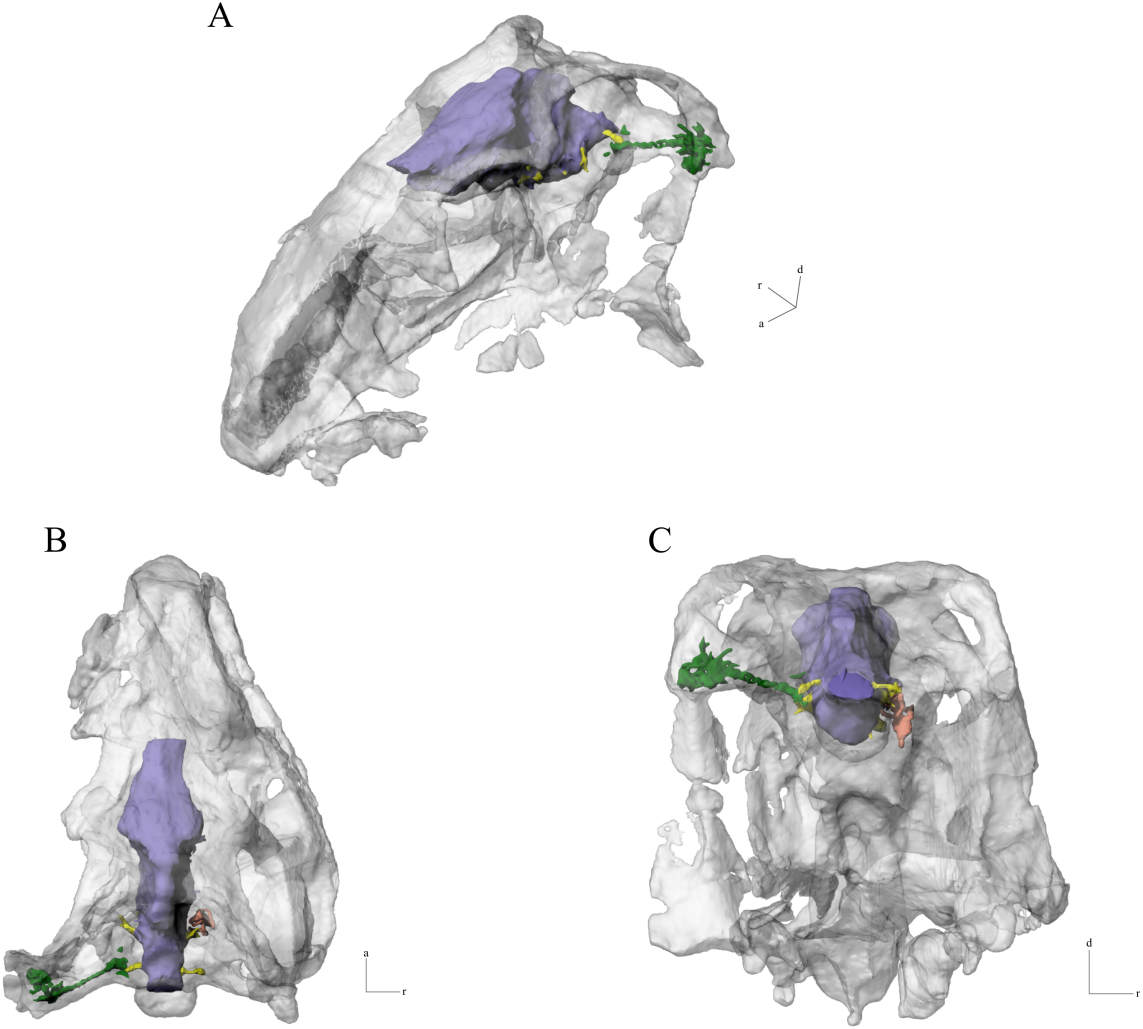
Endocast (blue), right inner ear (orange), and middle ear sinus system (green) of *N. engaeus* in the skull of the specimen PVL 5698. (A) Dorsolateral, (B) dorsal, and (C) posterior views. 3D reconstruction: JRA Taborda.

The middle ear sinus system could be identified on the left side of the digital endocast of *N. engaeus*, but no structures corresponding to the paratympanic and pharyngotympanic sinuses could be recognized (Fig. 5).

The middle ear sinus system pierces through the paroccipital process; it is a thin, straight, and tubular canal that is posterolaterally directed. It expands dorsoventrally on its distal end being four times higher than the proximal region (Figs. 5A–5C). This expansion corresponds to the cranioquadrate passage that would house the stapedial artery and a branch of the facial nerve (Bona, Paulina-Carabajal & Gasparini, 2017). The siphonium should be located ventral to this structure, but it could not be identified on this digital endocast, despite recognizing its distal exit on the quadrate foramen of PVL 5698.

## Comparison with Other Archosauriform Endocasts

**Figure 6:**
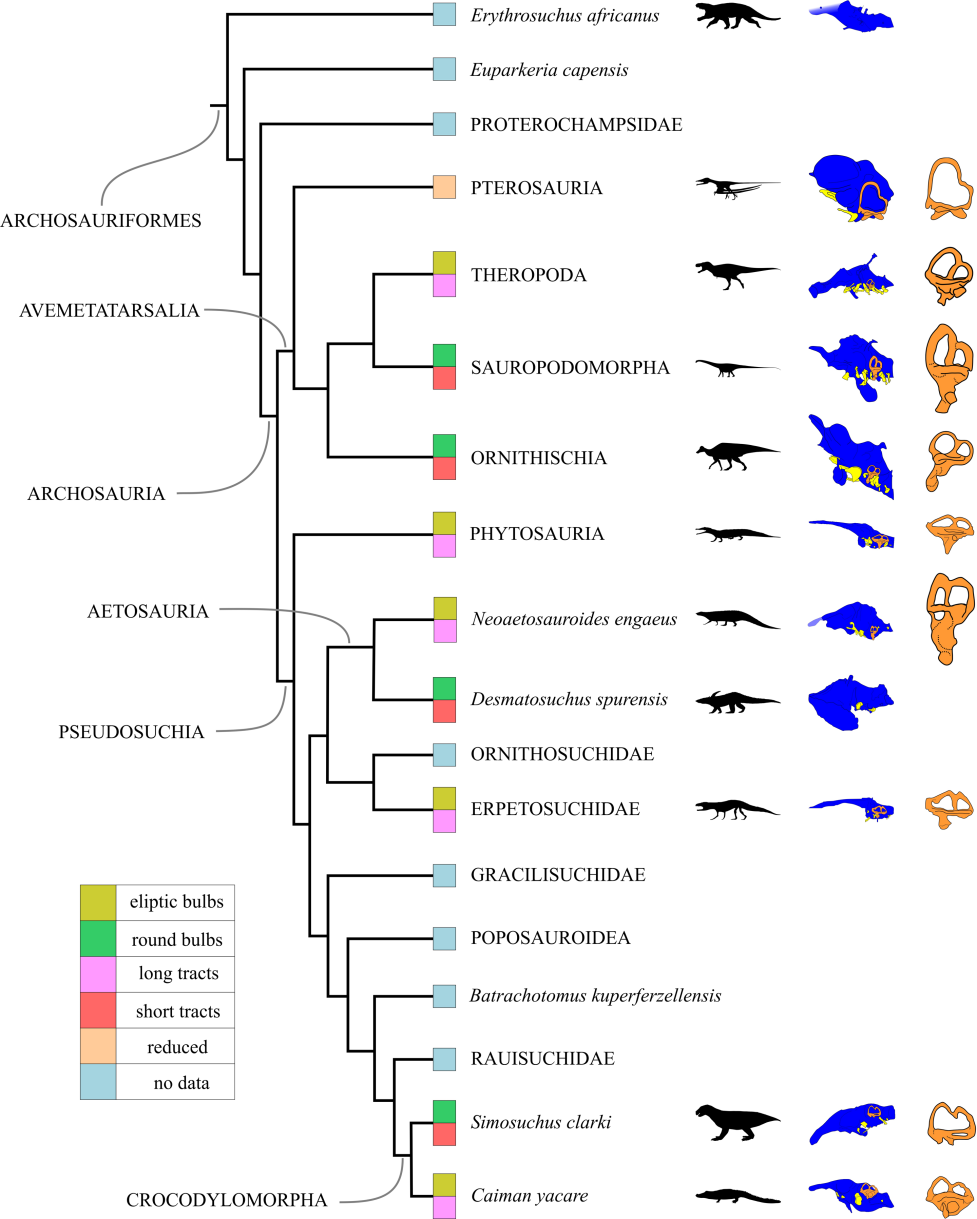
Endocranial anatomy of several archosauriform taxa and their phylogenetic relationship (phylogeny modified from Ezcurra et al., 2017). Life reconstructions of selected archosauriforms in black, encephalon in blue (not to scale), vestibular apparatus in orange, and cranial nerves in yellow. Olfactory region features plotted on the phylogeny, see references within figure. Archosauriform endocasts redrawn from Gower & Sennikov, 1996 (archosauriforms *Erythrosuchus africanus*); Witmer et al., 2003 (pterosaur *Anhanguera santanae*); Witmer & Ridgely, 2008 (theropod *Tyrannosaurus rex* and sauropod *D. longus*); Evans, Ridgely & Witmer, 2009 (ornithischian *H. altispinus*); Lautenschlager & Butler, 2016 (phytosaur *P. angustifrons*); Hopson, 1979 (aetosaur *D. spurensis*); Nesbitt et al., 2018 (erpetosuchid *P. gracilis*); Kley et al., 2010 (crocodylomorph *S. clarki*); MACN-HE 43694 (crocodilian *C. yacare*).

Comparing the endocast of *N. engaeus* with *D. spurensis* there are some noticeable differences between these aetosaurs, among archosauriforms (Fig. 6). The olfactory tracts of *N. engaeus* (PVL 5698, PULR 108) are narrow being half the width of the cerebral hemispheres, whereas the olfactory tracts of *D. spurensis* (UMMP 7476; Hopson, 1979) are about the same width of the cerebral hemispheres as it also happens in the erpetosuchid *Parringtonia gracilis* (Nesbitt et al., 2018). This condition of narrow olfactory tracts seen in *N. engaeus* resembles more that of archosauriforms (*Tropidosuchus romeri*: Trotteyn & Paulina-Carabajal, 2016; *Triopticus primus*: Stocker et al., 2016), phytosaurs (*Ebrachosuchus neukami, Parasuchus angustifrons*: Lautenschlager & Butler, 2016; *Wannia scurriensis*: Lessner & Stocker, 2017, ornithosuchids (*Riojasuchus tenuisceps*: Baczko, Taborda & Desojo, 2012), and crocodylomorphs (*Sebecus icaeorhinus*: Hopson, 1979; *Simosuchus clarki*: Kley et al., 2010; *Caiman yacare, Alligator mississippiensis,* and *Gavialis gangeticus*: Bona, Paulina-Carabajal & Gasparini, 2017), theropods (*Giganotosaurus carolinii*: Paulina-Carabajal & Canale, 2010; *Sinraptor dongi*: Paulina-Carabajal & Currie, 2012), ornithischians (e.g., *Corythosaurus* sp., *Hypacrosaurus altispinus*: Evans, Ridgely & Witmer, 2009; *Arenysaurus ardevoli*: Cruzado-Caballero et al., 2015; *Euoplocephalus tutus*: Miyashita et al., 2011; *Pachycephalosaurus wyomingensis*: Giffin, 1989, *Stegoceras validum*: Stocker et al., 2016), and sauropods (*Amargasaurus cazaui*: Paulina-Carabajal, Carballido & Currie, 2014; *Diplodocus longus*: Witmer & Ridgely, 2008).

The olfactory bulbs of *N. engaeus* (PVL 4363) are elongated, longer than wide, resembling the condition of some phytosaurs (*P. angustifrons*, *E. neukami*) and most crocodylomorphs (*S. icaeorhinus*, *C. yacare*, *A. mississippiensis*) excepting *S. clarki*, and some theropod dinosaurs (*G. carolinii*, *Tyrannosaurus rex*). They also differ from those of the aetosaur *D. spurensis*, and some sauropod and ornithischian dinosaurs (*D. longus*, *E. tutus*, *H. altispinus, S. validum*), where the olfactory bulbs are as long as wide.

The cerebral hemispheres are clearly recognized in *N. engaeus* (PVL 5698). They are approximately one-third (1/3) wider than the MVC, similar to the pseudosuchians *R. tenuisceps*, *S. clarki,* and *S. icaeorhinus*, and lambeosaurine ornithischians (*H. altispinus*, *Corythosaurus sp.*, *A. ardevoli*) differing from the condition seen in phytosaurs (*P. angustifrons*, *Ebrachosuchus neukami*), the crocodylomorph *Gryposuchus neogaeus*, the theropods *G. carolinii* and *Acrocanthosaurus atokensis*, the ankylosaurids *Euoplocephalus tutus*, *Tarchia teresae*, and *Talaurus plicatospineus*, in which the cerebral hemispheres are almost as wide as the MVC. The aetosaur *D. spurensis* shows a condition that is intermediate between that seen in the previous taxa and *N. engaeus*.

The flexure between the major axis of the forebrain and that of the midbrain measured for *N. engaeus* (135°) resembles that of *Riojasuchus tenuisceps* (130°: Baczko & Desojo, 2016) and *D. spurensis* (120°: UMMP 7476), being slightly lower in this last one. This flexure has a lower angle than that of crocodylomorphs (*G. neogaeus*, *G. gangeticus*, *S. clarki*) and phytosaurs (*P. angustifrons*, *E. neukami, W. scurriensis*) which is around the 150°, contrasting with the more acute angles registered in some dinosaurs such as *D. longus*, *A. atokensis*, and *Tyrannosaurus rex* (110°) (Fig. 6). This variability in the angle between the forebrain and midbrain could be related to the height of the braincase which is apparently higher in the later than in the pseudosuchians mentioned above. Moreover, within pseudosuchians the lowest values were registered in phytosaurs and extant crocodiles which have the flattest skulls.

The hypophysis of *N. engaeus* was not preserved in the natural endocast (PULR 108) but the base of this structure can be identified in the digital reconstruction of PVL 5698, based on its topology and size. However, the hypophysis is still incomplete in PVL 5698 and therefore it cannot be determined whether it is vertical as in phytosaurs (e.g., *Ebrachosaurus neukami*, *P. angustifrons*) and ornithischians (e.g., *H. altispinus*, *Corythosaurus sp.*, *A. ardevoli*, *Euoplocephalus tutus*, *Talaurus plicatospineus*, *Panoplosaurus mirus*) or posteroventrally directed as in most archosaurs (e.g., *A. mississippiensis*, *G. gangeticus*, *S. icaeorhinus*, *S. clarki*, *R. tenuisceps*, *T. rex*, *D. longus*) (Fig. 6). The artificial endocast of the aetosaur *D. spurensis* (UMMP 7476) preserved only the posterior part of the hypophysis and the anterior region is obscured by leakage of the resin between the bones of the braincase used during its preparation.

The small floccular lobes identified in *N. engaeus* can also be recognized in the aetosaur *D. spurensis* and the gracilisuchid *Gracilisuchus stipanicicorum* (Baczko et al., 2015). The floccular recess is also present in other pseudosuchians such as phytosaurs (e.g., *P. angustifrons*, *Parasuchus hislopi*, *E. neukami*) as well as in *Batrachotomus kupferzellensis* and *Postosuchus kirkpartricki* but in the latter two it is larger than that of *N. engaeus* (PVL 5698) (Baczko et al., 2015). On the other hand, in crocodylomorphs the floccular recess is barely noticeable (e.g., *S. clarki*) or completely absent (e.g., *S. icaeorhinus*, *C. yacare*, *Crocodylus niloticus*). In proterochampsid archosauriforms (e.g., *Tropidosuchus romeri*, *Pseudochampsa ischigualastensis*) (Trotteyn & Paulina-Carabajal, 2016) the floccular lobes are absent as well, but in the non-archosaurian archosauriforms *T. primus*, *Euparkeria capensis*, and *E. africanus* the floccular lobes can be clearly recognized (Gower & Sennikov, 1996; Sobral et al., 2016; Stocker et al., 2016). Among avemetatarsalians, a large floccular lobe can be recognized in the basal sauropodomorph *Saturnalia tupiniquim*, in theropod dinosaurs, and in ornithischian stegosaurs and ankylosaurids (Trotteyn et al., 2015; Bronzati et al., 2017; Paulina-Carabajal et al., 2017).

The olfactory bulbs of *N. engaeus* (PVL 4363) are elongated and dorsally rounded, differing notably from those of *D. spurensis* (UMMP 7476) which are broad and flat. The latter also resembles the condition of some sauropod and ornithischian dinosaurs (e.g., *D. longus*, *E. tutus*), and brevirostrine crocodylomorphs (i.e., *S. clarki*) which have large rounded olfactory bulbs and short tracts. The condition of elongated olfactory bulbs seen in *N. engaeus* resembles that of some phytosaurs (i.e., *E. neukami*, *P. angustifrons*), poposauroids (i.e., *Shuvosaurus inexpectatus*: Lehane, 2005), and crocodylomorphs (i.e., *S. icaeorhinus*, *A. mississippiensis*, *C. yacare*). The olfactory tracts of *N. engaeus* are narrow and longer than in *D. spurensis*, but not as long as in phytosaurs and longirostrine crocodylomorphs.

In *N. engaeus*, the branches of the trigeminal nerve (CN V) exit through a single passage resembling the condition of non-archosaurian archosauriforms (*Tropidosuchus romeri*, *T. primus*), sauropod and ornithischian dinosaurs, and most pseudosuchians (*Desmatosuchus spurensis*, *Riojasuchus tenuisceps*, *Parringtonia fragilis*, *Postosuchus kirkpatricki*, *S. icaeorhinus*, *C. yacare*, *Alligator mississippiensis*), excepting phytosaurs (*P. angustifrons*, *E. neukami*) (Lautenschlager & Butler, 2016). This condition differs from that of some pterosaurs and theropod dinosaurs in which the ophthalmic branch (CN V1) and the combined canal for the maxillary (CN V2) and mandibular (CN V3) branches split inside the endocranial cavity (Witmer et al., 2003; Witmer & Ridgely, 2008). Therefore *N. engaeus* presents the plesiomorphic condition of a single exit for the trigeminal nerve within archosauriforms.

The location of the CNs VII and VIII of *N. engaeus* differs from that proposed by Case (1921) for *D. spurensis*. The exits for these two cranial nerves are identified separately on the digital endocast of *N. engaeus* (PVL 5698), whereas in *D. spurensis* they were identified by Case (1921) as a single foramen posterior to the exit of the CN VI and anterior to a putative exit for the CN IX-XI.

The exit of the CNs IX-XI can be recognized as a single passage posterior to the endosseous labyrinth and splits into two branches. This reconstruction for *N. engaeus* is congruent with that of Case (1921) for *D. spurensis*, but differs from the interpretation of Hopson who pointed out one shared exit for the CN IX and X (Hopson, 1979: figure 9). The foramen indicated by the latter author would correspond to the exit of the CN XII in concordance with the original interpretation by Case (1921) and the morphology of the digital endocast of *N. engaeus* (PVL 5698). These aetosaurs share the condition of a single exit for the CN XII with the archosauriforms *Euparkeria capensis* (Sobral et al., 2016) and *E. africanus* (Gower & Sennikov, 1996), phytosaurs (*E. neukami*, *P. angustifrons*: Lautenschlager & Butler, 2016) pterosaurs (*Allkaruen koi*: Codorniú et al., 2016) and theropods (*Tyrannosaurus rex*: Witmer & Ridgely, 2008; *G. carolinii*: Paulina-Carabajal & Canale, 2010). This pattern differs from that of the archosauriform *T. primus* (Stocker et al., 2016), crocodylomorphs (*S. icaeorhinus*: Hopson, 1979; *Simosuchus clarki*: Kley et al., 2010; *C. yacare*: MACN-HE 43694; *A. mississippiensis*: OUVC 9761; *G. gangeticus*: Bona, Paulina-Carabajal & Gasparini, 2017), ornithischians *A. ardevoli*: Cruzado-Caballero et al., 2015; *Anchiceratops ornatus:* (Hopson, 1979); *T. plicatospineus*: Paulina-Carabajal et al., 2017), and sauropodomorphs (*Diplodocus longus*: Witmer et al., 2008; *S. tupiniquim*: Bronzati et al., 2017) in which the hypoglossal nerve exits through multiple foramina.

The size and location of the CNs II, III, IV and VI do not exhibit any particular difference between *N. engaeus* and *D. spurensis*.

The endosseous labyrinth of *N. engaeus* is almost as dorsoventrally high as anteroposteriorly long and the internal diameter of the anterior semicircular canal is equivalent to the posterior one (Fig. 4). This last condition is also present in the pseudosuchians *D. spurensis*, *P. angustifrons*, *E. neukami*, *P. gracilis*, and the ornithischian dinosaurs *E. tutus* and *A. ornatus*, and titanosaurid sauropod dinosaurs (Hopson, 1979; Knoll et al., 2015; Stocker et al., 2016; Lautenschlager & Butler, 2016; Nesbitt et al., 2018) (Fig. 6).

The angle formed between the anterior semicircular canal with the common crus, and the posterior semicircular canal with the later are similar, more than 45° from the common crus plane in *N. engaeus* (Fig. 4). This morphology can also be seen in *Chanaresuchus bonapartei*, some phytosaurs (i.e., *P. angustifrons*, *E. neukami*), *G. stipanicicorum*, *Parrintonia fragilis*, many crocodylomorphs (i.e., *G. neogaeus*, *A. mississippiensis*, *G. gangeticus*), and ornithischian dinosaurs (*E. tutus*, *A. ornatus*, *Pachycephalosaurus wyomingensis*) (Hopson, 1979; Witmer & Ridgely, 2008; Lautenschlager & Butler, 2016; Stocker et al., 2016; Bona, Paulina-Carabajal & Gasparini, 2017; Nesbitt et al., 2018). This condition differs from the archosauriform *Triopticus*, the loricatan *Postosuchus kirkpatricki*, some crocodylomorphs (i.e., *S. clarki*), pterosaurs, theropod and most sauropodomorph dinosaurs (*S. tupiniquim*, *D. longus*, *Allosaurus fragilis*, *T. rex*) (Witmer & Ridgely, 2008; Kley et al., 2010; Codorniú et al., 2016; Stocker et al., 2016) in which the anterior semicircular canal is generally taller dorsoventrally than the posterior because the anterior canal branches taller than the common crus forming a very acute angle (less than 45° from the crus plane). An intermediate condition can be recognized in the aetosaur *D. spurensis* (Stocker et al., 2016) in which the anterior canal branches at 45° from the common crus.

The angle formed between the anterior and posterior semicircular canals is highly variable among archosauriforms being less than 90° in a wide variety of taxa including *N. engaeus*, *P. gracilis*, *Junggarsuchus sloani*, *T. primus, S. tupiniquim*, *A. koi*, *D. longus*, and *T. rex* (Georgi & Sipla, 2008; Witmer & Ridgely, 2008; Codorniú et al., 2016; Stocker et al., 2016; Bronzati et al., 2017; Nesbitt et al., 2018). On the contrary, the angle is approximately 90° in phytosaurs (*E. neukami*, *P. angustifrons*), crocodylomorphs (*G. neogaeus*, *C. yacare* (MACN-HE 43694), *A. mississippiensis* (OUVC 9761), *Gavialis gangeticus*, *C. niloticus* (Lautenschlager & Butler, 2016; Bona, Paulina-Carabajal & Gasparini, 2017), and several ornithischians (*H. altispinus*, *Lambeosaurus sp.*: Evans, Ridgely & Witmer, 2009).

The lagena is almost dorsoventrally subequal to the vestibulum in *N. engaeus* (Fig. 4) as well as in *D. spurensis*. Unlike aetosaurs, the lagena is dorsoventrally longer than the vestibulum in phytosaurs, *C. niloticus,* several ornithischians (*H. altispinus*, *Lambeosaurus sp.*, *Euoplocephalus tutus*), and *T. rex*, and it is even shorter than the vestibulum in *Chanaresuchus bonapartei*, *P. gracilis*, *A. mississippiensis*, and *D. longus* (Witmer & Ridgely, 2008; Witmer & Ridgely, 2008; Evans, Ridgely & Witmer, 2009; Lautenschlager & Butler, 2016; Stocker et al., 2016; Nesbitt et al., 2018). The lagena is reduced in *G. neogaeus*, *Gavialis gangeticus*, and pterosaurs (*A. koi*, *Anhanguera santanae*) (Witmer et al., 2003; Codorniú et al., 2016; Bona, Paulina-Carabajal & Gasparini, 2017).

## Discussion

When analyzing the olfactory region, two general patterns could be recognized. The first corresponding to short olfactory tracts and wide, rounded bulbs that was seen in archosaurs associated to herbivorous habits such as *D. spurensis*, *S. clarki*, *Stegosaurus armatus*, *E. tutus*, *S. validum*, *H. altispinus*, *Corythosaurus sp.*, and *D. longus*. On the other hand, the other pattern with elongated tracts and narrow, elliptic bulbs was present in archosaurs associated to carnivorous habits like *P. angustifrons*, *P. gracilis*, *S. icaeorhinus*, *C. yacare*, *Sinraptor dongi*, *Carcharodontosaurus saharicus*, *A. atokensis*, and *T. rex* (Fig. 6). *N. engaeus* has a morphology that resembles the second pattern corresponding to carnivorous habits, which is also congruent with the dental morphology of this aetosaur (Desojo & Báez, 2007).

Several authors discussed the orientation of the lateral semicircular canal of the inner ear proposing that in an ‘alert’ or ‘neutral’ position this canal should be parallel to the ground (e.g. Witmer et al., 2003; Sereno et al., 2007; Witmer & Ridgely, 2008). When orienting the skull of *N. engaeus* with the lateral semicircular canal parallel to the ground, the snout is anteroventrally directed and its ventral margin forms an angle of approximately 30 degrees with the horizontal plane. This position favours both a more binocular visibility, with the snout being less obstructive for the visual field, as well as the nares positioned towards the ground for tracking. However, different studies propose that the lateral semicircular canal should not be used as a reference for the skull orientation based on documented evidence on living tetrapods whose semicircular canals tend to be misaligned with the Earth’s axes (Hullar, 2006; Taylor, Wedel & Naish, 2009; Marugán-Lobón, Chiappe & Farke, 2013). The misalignment of the semicircular canals is apparently physiologically advantageous to perceive the angular acceleration in all canals during horizontal head rotation (Cohen & Raphan, 2004). Discarding the lateral semicircular canal as a reference system, other structures need to be considered when orienting the skull. If the maxillary tooth row parallel to the ground is used to orient the skull (Marugán-Lobón, Chiappe & Farke, 2013), the occipital region of *N. engaeus* faces posteroventrally and it adopts an antinatural position when trying to articulate the skull with the cervical osteoderms of the dorsal armour of this aetosaur as well as when articulating the occipital condyle with the atlas. Following the discussion presented by Kley et al. (2010) for the pseudosuchian *S. clarki*, the central portion of the palate and ventral surface of the braincase can be used as reference to orient the posture of the head. When orienting the skull of *N. engaeus* with the palate and ventral surface of the braincase, the lateral semicircular canal is almost horizontal, tilted slightly anterodorsally at five degrees from the ground, whereas the snout is oriented anteroventrally at approximately 27 degrees from the horizontal (Fig. 3A).

Holding the skull in this position and the morphology of the olfactory region, different from that of an herbivorous aetosaur (*D. spurensis*), would support the proposal of animalivorous habits for this aetosaur. These animalivorous habits for *N. engaeus* were previously proposed by Desojo & Vizcaíno (2009) based on its jaw biomechanics and dental morphology. The conical teeth of *Neoaetosauroides engaeus* do no exhibit wear facets, differing from the leaf-shaped, serrated teeth with wear facets of several aetosaurs that were better adapted for crushing, chopping, and slicing and evidenced some degree of food processing capacities. The jaw biomechanics of *N. engaeus* allowed inferring bite movements faster than those of herbivorous aetosaurs (Desojo & Vizcaíno, 2009). These fast bite movements suggested for *N. engaeus* could allow catching prey such as invertebrates and microvertebrates, a condition consistent with the neuroanatomical features discussed above. Therefore, *N.* would occupy a different role within Triassic continental communities, representing animalivorous habits probably alongside with other aetosaurs such as *Longosuchus meadei*, *Aetosaurus ferratus*, *Aetosauroides scagliai* and *Paratypothorax sp*. based on their cranial morphology (Desojo & Báez, 2005; Desojo & Báez, 2007). This differs from the traditional herbivorous condition proposed for aetosaurs, which is generally based on the northern hemisphere taxa, such as *D. spurensis* and *Stagonolepis robertsoni* (Walker, 1961; Small, 2002; Desojo et al., 2013).

## Conclusion

Here we carried out the first description of a natural endocast of an aetosaur, *N. engaeus*, and also developed the first digital endocast which complemented the former including delicate structures such as the inner ear and the middle ear sinus. The anatomy of the endocast of *N. engaeus* exhibited remarkable differences (e.g., location of CNs VII, VIII, XII) when compared with the neuroanatomical features previously known for aetosaurs, based only in *D. spurensis* (Case, 1921; Hopson, 1979; Stocker et al., 2016). The new neuroanatomical information provided by this contribution allowed us to make an incursion about the paleobiology of *N. engaeus* supporting this taxon as an animalivorous aetosaur, which differs from the traditional interpretation of aetosaurs as exclusively herbivorous pseudosuchians.

These results contribute to the knowledge of aetosaurs and their paleoneurology, a topic poorly studied among pseudosuchians but crucial for the understanding of archosaur paleobiology and evolution.

## Institutional Abbreviations

MACN-HE: Museo Argentino de Ciencias Naturales “Bernardino Rivadavia”, Colección Herpetología, Buenos Aires, Argentina
OUVC: Ohio University, Vertebrates Collection, Ohio, USA
PULR: Museo de Paleontología, Universidad Nacional de La Rioja, La Rioja, Argentina
PVL: Instituto Miguel Lillo, Paleontología de Vertebrados, Tucumán, Argentina
UMMP: University of Michigan Museum of Paleontology, Ann Arbor, Michigan, USA

##  Supplemental Information

10.7717/peerj.5456/supp-1 Supplemental Information 1 3D pdf of the skull of *Neoaetosauroides engaeus* based on the specimens PVL 4363 and PVL 5698 Fossil skull in brown, encephalon in blue, inner ear in orange, cranial nerves in yellow, and middle ear sinus system in green.

10.7717/peerj.5456/supp-2 Supplemental Information 2 ‘Alert’ or ‘neutral’ orientation of the skull of *Neoaetosauroides engaeus* based on the digital reconstruction of PVL 5698 and PVL 4363 skulls overlapped Black line and **dotted line,** horizontal plane; **green line,** main axis of the lateral semicircular canal; **yellow line,** snout orientation. The angles between the ventral surface of the braincase and palate with the lateral semicircular canal and that with the snout inclination and are indicated.

## Additional Information and Declarations

### Data Availability

The following information was supplied regarding data availability: Taborda, Jeremías (2018): Neoaetosauroides engaeus (PVL 5698) - CTscan. figshare. Figure. https://doi.org/10.6084/m9.figshare.6709289.v1. This dataset is under embargo until June 27th, 2019 to coordinate with the museum which will be housing both the CT scan images along with the collections. Both will be publicly accessible for any researcher by request submitted to the curator (Dr. Pablo E. Ortiz, from the Instituto Miguel Lillo, Tucumán, Argentina; peortizchamb@gmail.com).
