# Supplementary figures and images for: Paleoneuroanatomy of the aetosaur Neoaetosauroides engaeus (Archosauria: Pseudosuchia) and its paleobiological implications among archosauriforms
Source: PeerJ. 2018 Aug 22;6:e5456. doi: 10.7717/peerj.5456 (PMC6109373; doi:10.7717/peerj.5456)

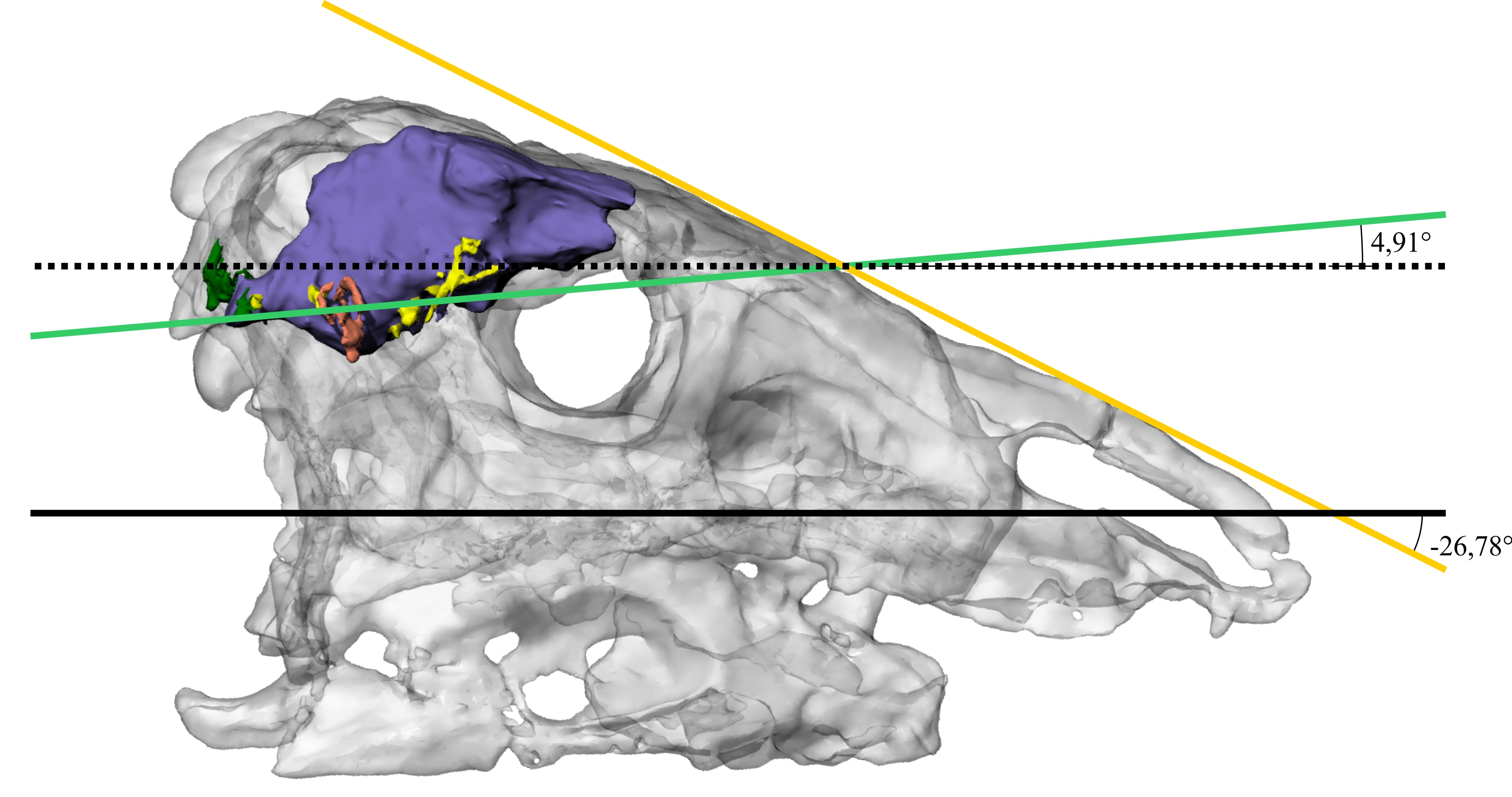

Supplement: Supplemental Information 2 — Black line and dotted line, horizontal plane; green line, main axis of the lateral semicircular canal; yellow line, snout orientation. The angles between the ventral surface of the braincase and palate with the lateral semicircular canal and that with the snout inclination and are indicated. [file peerj-06-5456-s002.jpg]
